# Alginates along the filament of the brown alga *Ectocarpus* help cells cope with stress

**DOI:** 10.1038/s41598-019-49427-z

**Published:** 2019-09-10

**Authors:** Hervé Rabillé, Thomas A. Torode, Benoit Tesson, Aude Le Bail, Bernard Billoud, Elodie Rolland, Sophie Le Panse, Murielle Jam, Bénédicte Charrier

**Affiliations:** 1 CNRS, Sorbonne Université, Laboratoire de Biologie Intégrative des Modèles Marins LBI2M, Station Biologique, Roscoff, France; 2 The Sainsbury Laboratory, University of Cambridge, Bateman Street, Cambridge, United Kingdom; 3 Marine Biology Research Division, Scripps Institution of Oceanography, University of California San Diego, La Jolla, CA USA; 4 Platform Merimage, FR 2424, CNRS, Station Biologique, Roscoff, France; 5 Marine Glycobiology team, UMR8227, CNRS-UPMC, Station Biologique, Roscoff, France; 6 Department of Cell Biology, Friedrich-Alexander-University of Erlangen-Nürnberg, Erlangen, Germany

**Keywords:** Cell biology, Ocean sciences

## Abstract

*Ectocarpus* is a filamentous brown alga, which cell wall is composed mainly of alginates and fucans (80%), two non-crystalline polysaccharide classes. Alginates are linear chains of epimers of 1,4-linked uronic acids, β-D-mannuronic acid (M) and α-L-guluronic acid (G). Previous physico-chemical studies showed that G-rich alginate gels are stiffer than M-rich alginate gels when prepared *in vitro* with calcium. In order to assess the possible role of alginates in *Ectocarpus*, we first immunolocalised M-rich or G-rich alginates using specific monoclonal antibodies along the filament. As a second step, we calculated the tensile stress experienced by the cell wall along the filament, and varied it with hypertonic or hypotonic solutions. As a third step, we measured the stiffness of the cell along the filament, using cell deformation measurements and atomic force microscopy. Overlapping of the three sets of data allowed to show that alginates co-localise with the stiffest and most stressed areas of the filament, namely the dome of the apical cell and the shanks of the central round cells. In addition, no major distinction between M-rich and G-rich alginate spatial patterns could be observed. Altogether, these results support that both M-rich and G-rich alginates play similar roles in stiffening the cell wall where the tensile stress is high and exposes cells to bursting, and that these roles are independent from cell growth and differentiation.

## Introduction

The brown algae (Phaeophyceae) belong to the Stramenopiles, which have been phylogenetically separated from other plant and algal lineages for ~1.5 billion years^1^. They are thought to have arose ~200 mya^2^ making it a relatively recent event in comparison to the colonisation of land and diversification of land plants ~510–630 mya^3,4^. In this time the brown algae have independently evolved complex multicellularity and have diversified into the largest and most morphologically intricate group of the macroalgae. The unique evolutionary history of brown algae and their lifestyle in a marine ecosystem (characterised by strong abiotic stresses) make them likely to have acquired original cellular and biophysical mechanisms during development^5^. Cell walls are of vital importance for the structure and shape of plants and algae, and provide the first line of defence against abiotic stress. The cell walls of brown algae are different in both composition and abundance of cell wall components from that of land plants and other macroalgae^6,7^. Cellulose microfibrils are less abundant in brown algal cell walls, accounting for only 1–8% of the cell wall dry weight^8^. Consequently, the cell walls of brown algae are more elastic than that of land plants^9^, having not evolved to resist compressive forces required for terrestrial growth. The major components of brown algal cell walls are sulphated fucans (~40%) and alginates (~40%) which are anionic polysaccharides. The walls also contain proteins, arabinogalactan proteins, phlorotannins (halogenated or sulphated phenolic compounds) and iodine^10,11^. In land plants, mechanical properties of cell walls are largely modulated by the pectic hydrogel matrix which is composed of multiple sub-families of polysaccharides of diverse sugar composition and structure^12–14^. In brown algae, the hydrogel matrix is composed of alginate which is a linear polysaccharide composed of β-1,4-D-mannuronate (M) and α-1,4-L-guluronate (G). Alginate is produced as pure mannuronate, and is converted into guluronate via mannuronan-C5-epimerases (MC5Es). The activity of the MC5E family leads to the generation of three distinct regions within the alginate structure, homopolymer blocks of mannuronan (M-blocks) or guluronan (G-blocks), and heteropolymer regions of interspersed M and G (MG-blocks).

The G-block regions are able to form “egg-box” cross-links via calcium, and alginate gels made *in vitro* show that their viscosity depends on the M/G ratio and more specifically on the presence of G-blocks^15–17^. This is analogous to de-methylated stretches of homogalacturonan which allow calcium cross-linking in land plants. However, whereas de-methylation allows access of calcium ions to the homogalacturonan backbone, the conversion of mannuronate to guluronate in alginate causes a conformational change in the sugar residue resulting in an altered secondary structure in the alginate backbone. This causes a unique combination of sugar linkages whereby M-blocks are connected by diequatorial linkages, whilst G-blocks are connected diaxially and form strong intra-molecular hydrogen bonds. MG-blocks contain both diequatorial and diaxially linked residues. The modified secondary structure alters the flexibility of the different blocks of the alginate polysaccharide, with MG being the most flexible and GG the most rigid (flexibility: MG > MM > GG)^18^. Interestingly, the secondary structure of MG-blocks allows formation of calcium cross-linking, but has a lower affinity for calcium compared to the G-blocks^19,20^, allowing for a two-tier hierarchical structure of calcium cross-linking within a single polysaccharide structure.

Furthermore, alginate has recently been reported to form tertiary microfibrils structures of ~4 nm diameter within the cell wall of brown algae^21^. In the brown alga *Ectocarpus* the cell wall of the prostrate sporophyte filaments lacks any apparent specific organisation^22,23^. However, tomography performed on upright filaments showed that cellulose microfibrils adopt an isotropic organisation, whereas alginate microfibrils assemble into a cross-linked network mainly in the z-axis^21^. This suggests that the alginate microfibrils function to constrain deformation of the cell wall in the z-axis, thereby maintaining the cell wall isotrope transversally. Additionally, the alginate matrix may be fortified via the addition of phlorotannins^24^. The formation of a covalently bound alginate-phlorotannin network stabilises the alginate matrix and provides an alternative to ionically cross-linking via calcium. Incorporation of phlorotannins into the wall can occur naturally over development^25^, and also during wounding responses^26,27^.

Whilst the mechanical roles of alginate gels have been widely studied *in vitro*, this is not directly informative about the role of alginates within cell walls. Indeed, previous research into the composition of alginates within tissues has demonstrated an opposite relationship between the ratio of G-rich alginates (shown to be the most rigid *in vitro*) and the stiffness of the algal tissue (e.g.^28^).

Recently, monoclonal antibodies have been raised against different blocks of alginates^29^, allowing for spatial analysis of alginate distribution *in situ*. Using these antibodies in the large alga *Sargassum*, M-rich alginates were immunolabeled in mature tissues while enrichment in G-units was observed in the quite quiescent, central cell of the apical meristematic region^30^. In the brown alga *Fucus*, M-rich, MG-rich and G-rich alginates were all labelled in the zygote with a similar, ubiquitous pattern, and became undetectable in the growing rhizoid^29^. Therefore, these first studies do not allow to infer a general pattern and roles of alginate and of its different conformation, within brown algal tissues.

*Ectocarpus* is a filamentous alga that is easily cultivable and amenable to experimental manipulation. Initial vegetative growth consists of filaments that can attach and grow on a variety of laboratory equipment (e.g. cover slips, slides)^31,32^. In addition, because its filaments are uniseriate, modification of the growth conditions impacts all cells, allowing an easier interpretation of cell responses to external cues. Finally, prostrate filaments differentiate distinct cell types displaying different cell shapes and developmental fates^31^. This makes *Ectocarpus* an interesting model organism where cell chemistry, mechanics and shape can be studied in the frame of a whole organism.

In this study, we assessed the importance of alginates in regulating mechanical properties along the developing prostrate filament of *Ectocarpus* sporophytes by 1) immunolocalising the different alginate blocks and 2) looking for concomitant alterations to cell wall mechanical properties.

## Results

### Cell-specific pattern of alginate occurrence along the filament of *Ectocarpus*

**Figure 1:**
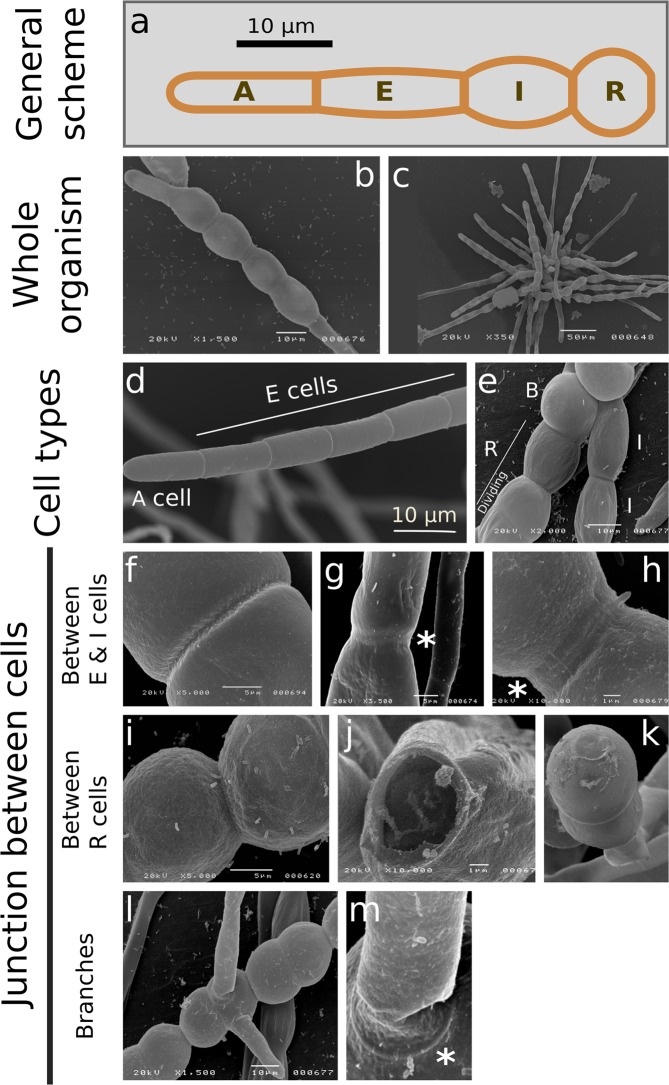
Filament organisation and cell morphologies observed by scanning electronic microscopy. (**a**) Overview of *Ectocarpus* sporophyte filament (prostrate) growing from spore germination. Five cell types are defined according to their position and shape. A type: Apical cell; E type: Elongated, cylindrical cell; I type: Intermediate cell; R type: Round, spherical cells positioned at the central region of the filaments; B type: Branched cells. Cell types are defined according to their position (for A cells) and their ratio of their length (L) to their width (w) (E, I and R cells). E cell: L/w > 2; I cell: L/w in [1.2; 2[; R cell: L/w < 1.2. The number of E, I, R and B increases with the filament maturation stage. Cells of the same cell types are contiguous. (**b**,**c**) Whole organism observed by scanning electronic microscopy (SEM); One week post germination (**b**), or 2–3 weeks post germination (**c)**.**(d**) A and E cells at the filament extremity. (**e**) I and R cell types in the central region of the filament. B indicates branching cells. (**f**–**h**) Junctions between E cells (**f**) and I cells (**g**,**h**), showing either single- (**f**) or double- ring(s) framing the wall (asterisks in **g**, **h**). (**i**–**k**) Junctions between R cells. (**l**–**m**) Higher magnification on branches, showing a ring at the junction site (asterisk).

In the early developmental stages (prostrate), *Ectocarpus* filaments grow as a string of cells generated from elongation and division of the highly polarised apical cell (A cell; Fig. 1a,b). Sub-apical cylindrical cells (E cells) progressively differentiate into spherical cells (R cells)^33^. As a result, the centre of the filament is mainly composed of spherical cells (Fig. 1b,c), which are also sites for the initiation of branches^33^ (Fig. 1c).

Branches repeat the same series of cell events, leading rise to a tuft of filaments after ~4 weeks (Fig. 1c). In-detail observation of the prostate filament by scanning electronic microscopy (SEM) revealed a homogeneous and fairly smooth surface along A and E cell types (Fig. 1d,f), and a more granular surface in I, R and B cell types (Fig. 1e,g,h,i). The presence of a ring structure of unknown nature was noticed at the junctions between I and R cells (Fig. 1g–k), as well as at the branching site (Fig. 1m). In some I and R cell types, it was possible to distinguish a double ring within this structure (Fig. 1h).

**Figure 2:**
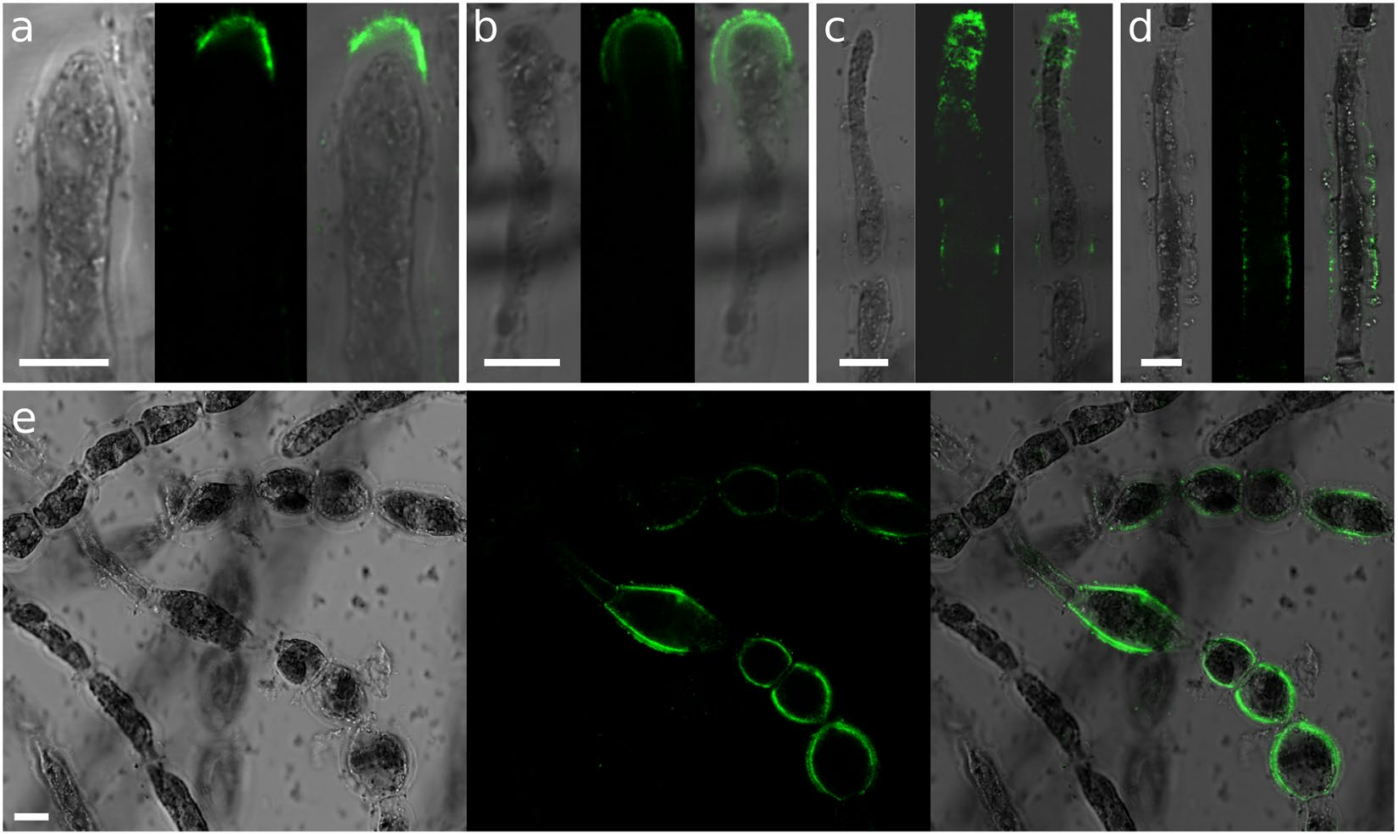
Mannuronate-rich alginate regions labelled with BAM6 antibody. BAM6 labelling of: (**a**–**c**) A cells; (**d**) E cells; (**e**) I and R cell types. Bright field, confocal and merge channels are shown for each cell. Acquisition time and laser intensity were constant. Scale bar 10 µm.

In order to map the distribution of alginates along the growing filaments, we whole-mount immunolabelled filaments using a sub-set of the brown algal monoclonal (BAM) series of monoclonal antibodies (MAbs) which recognise specific structural conformation of alginates^29^. BAM6 antibody recognises regions of alginate rich in mannuronans (M regions). When used in *Ectocarpus*, BAM6 mainly labelled the dome of the apical cells (Fig. 2a–c) and the shanks of the R and I cells (Fig. 2e). In A cells, labeling was most frequent in the most distal half (Fig. 2a) or the whole dome (Fig. 2b). However, in some cases, labeling extended across the entire dome and down the shanks (Fig. 2c), and in rare cases, shanks of some E cells were weakly labelled (Fig. 2d).

**Figure 3:**
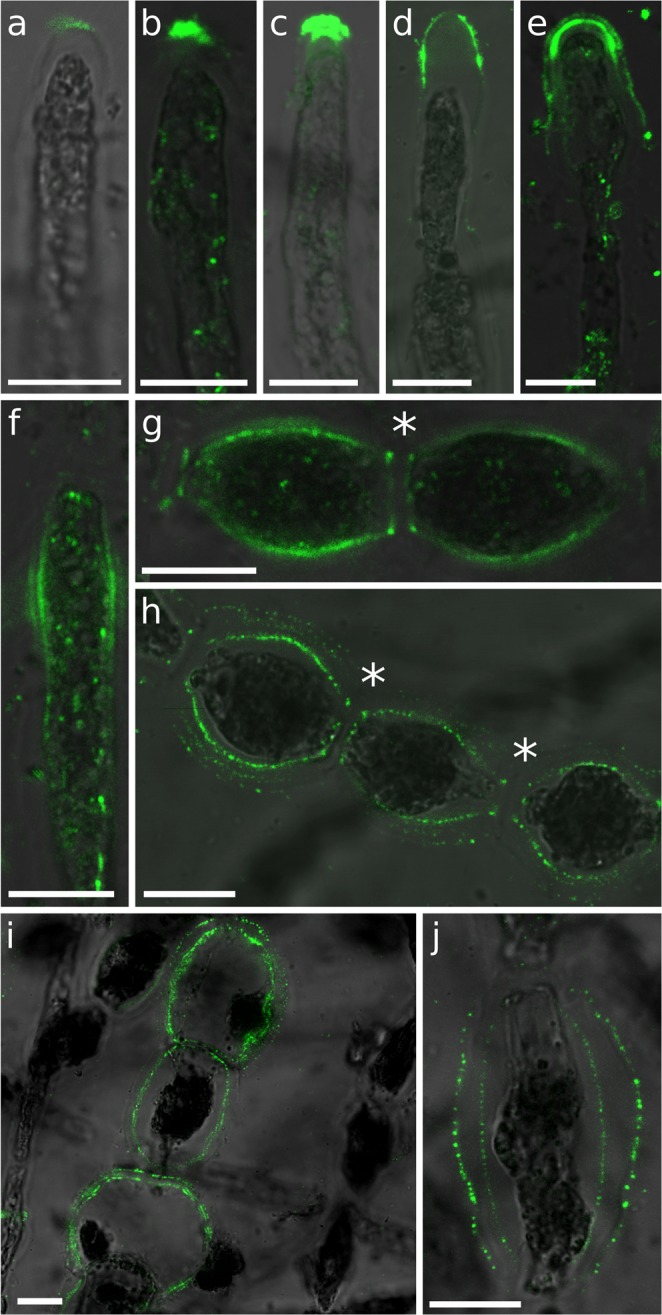
Mannuronate-Guluronate alginate regions labelled with BAM7 antibody. BAM7 labelling of: (**a**–**e**) A cells; (**f**) E-cell; (**g**–**j**) I and R cells. Merge of bright field and fluorescent signals are shown. Fluorescent signal was acquired with different acquisition times depending on the micrograph. Asterisk indicates the double rings. Scale bar 10 µm.

Enrichment of the cell wall with guluronate residues was assessed using the monoclonal antibody BAM7, which recognies M-G regions of alginate^29^. BAM7 labelled mainly the dome of the apical cell, in regions ranging from the tip (Fig. 3a–c) to an extended area including the whole dome up to the very adjacent shanks (Fig. 3d,e). Interestingly, in some instances, labelling of two distinct cell wall layers could be observed (Fig. 3c,e). E cells transitioning into I-type (i.e. rounding) also displayed slight signal in the center of their longitudinal surface, corresponding to the most curved regions in the longitudinal axis (Fig. 3f). In the central part of the filaments, both I and R cells were labelled (Fig. 3g,h), with R cells displaying the strongest signal (Fig. 3i). Two layers within the cell wall were clearly displayed (Fig. 3j). Another interesting observation was the presence of two rings framing the transverse separation between two adjacent cells (Fig. 3g,h, asterisks).

**Figure 4:**
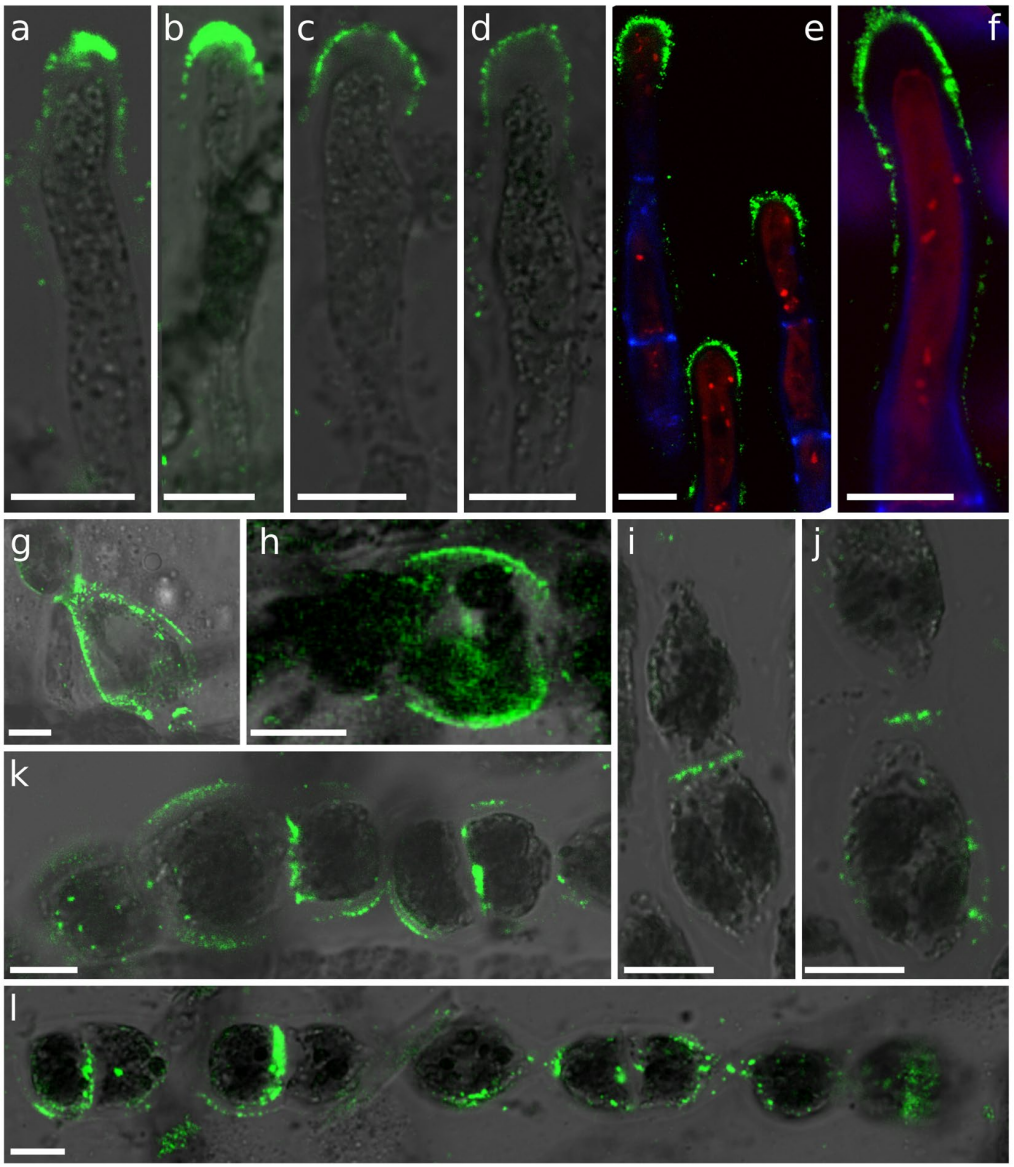
Guluronan-rich alginate regions labelled with BAM10 antibody. BAM10 labelling of: (**a**–**f**) A cells; (**h**–**j**) I cells; (**g**–**l**) R cells. Merge of bright field and fluorescent signals are shown, except in E & F where FITC, calcofluor (UV light, blue) and autofluorescence of chloroplasts (red) were merged. Fluorescent signal was acquired with different acquisition times depending on the photo. Scale bar 10 µm.

BAM10 binds G-rich regions in mixed MG alginates (e.g. GMGGGM)^29^. Its labelling was most intense in A cells. Different patterns were observed, ranging from regions located exclusively at the tip (Fig. 4a) to extended regions encompassing the whole dome (Fig. 4b,c), and even larger areas overlapping the adjacent shanks (Fig. 4d–f). In rare instances, signal could be observed in the curved shanks of I and R cells (Fig. 4g,h, respectively). BAM10 also distinctly labelled junctions between I cells (Fig. 4i,j) and showed a more heterogeneous distribution in R cells (Fig. 4k,l). Interestingly, BAM10 displayed the lamella between I cell transverse cell wall (Fig. 4i,j), whereas BAM7 labelled the two opposing transverse cell walls (Fig. 3g,h).

**Figure 5:**
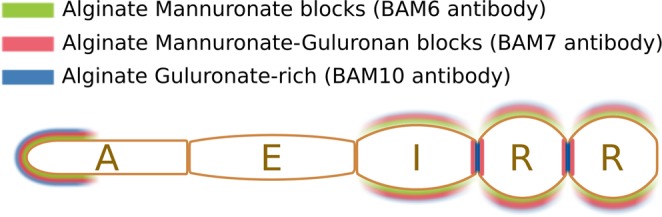
Summary of alginate mapping along the filament of *Ectocarpus*. Schematic of a sporophyte filament is shown with the four main cell types A, E, I and R. In living filaments, several cells of each type are grouped together. For simplicity, only one cell of each cell type is shown here. Colours indicate immunolocalisation of monoclonal antibodies BAM6, BAM7 and BAM10. Colour lines do not represent different cell wall layers, but the different antibodies.

In summary, the recognition of distinct alginate epitopes by BAM6, BAM7 and BAM10 displayed strong and overlapping distribution at the apical cells, whereas mature cells have distinct spatial patterning of alginates. All three MAbs show variability in the spatial distribution observed in the different apical cells, suggesting extensive re-modelling of alginate at the growing tips (Fig. 5). In mature cells, clear differences were observed in the shanks of intermediate (I) or round (R) cells which are enriched in mannuronans, while the transverse junctions between adjacent cells are enriched in guluronans.

### The alginate pattern is not correlated to cell wall maturation and cell wall thickness

**Figure 6:**
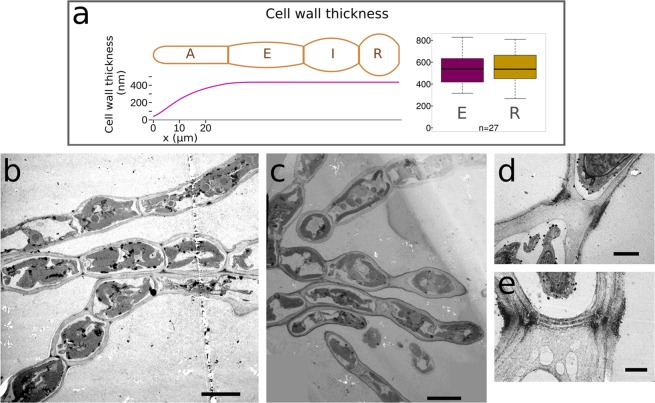
Cell wall thickness and structure along the filament. (**a**) (Right) Quantification of cell wall thickness in E and R cell types from TEM micrographs (n = 27, Student t-test, P value = 0.867), and (Left) Schematic representation summarising the cell wall thickness (y-axis, in nm) in different cell types along the filament (x-axis, in µm). (**b**,**c**) Representative TEM longitudinal sections showing uniform cell wall organisation and thickness along the filaments. (**d**,**e**) Higher magnification of transverse junctions showing the presence of dark rings. Scales 10 µm (**b**,**c**), 2 µm (**d**) or 1 µm (**e**).

To further explore the role of alginates in *Ectocarpus*, we searched for a functional relationship between the observed immunostaining pattern and the cell wall morphology. In order to check for different cell wall structures between cell types, sections of filaments were observed by TEM. Uniquely, the cell wall of the apical cells features a gradient in thickness ranging from ~40 nm at the tip to ~400 nm at the shanks^22^ (Fig. 6a,b). Despite this variation in cell wall thickness along the filament, similar BAM labelling intensity was observed in both the apical cell tip and the shanks of R cells, where it is at least 10 times thicker. Therefore, cell wall thickness cannot be directly linked to the abundance of the BAM antibody signal. In addition, all the cells of the filament (except the tip of the A cells, because of the cell wall thinness) display a cell wall with a bilayer organisation, made of a thick inner layer and a darker, thinner outer layer (Fig. 6c). This organisation is similar to that reported in previous studies^34^. Consequently, the distribution of alginate cannot be directly linked to the thickness or to the ultrastructure of the cell wall within the filament cells. Finally, it cannot be due to different cell wall maturation level, because labelling is observed in both A cell tips and R cells, which are respectively the youngest and oldest cells of the filament.

TEM also revealed deposits of dark cell wall material frequently observed at the transverse junctions (Fig. 6d,e), reminiscent of the MG and G-rich alginate rings displayed by immunostaining (Figs 3 and 4).

### Alginates accumulate in areas subject to high wall stresses

**Figure 7:**
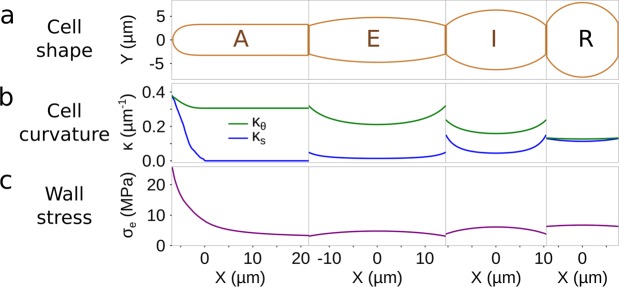
Curvature and wall stress along the filament. Curvatures Κ (y-axis, in µm^−1^) were calculated for each cell shape (Y) along the filament (X, in µm) in two perpendicular directions: the meridional (blue) and the circumferential (green) directions. Wall stress σ_e_ (y-axis, in MPa) was calculated by taking into account the turgor measured by limit plasmolysis (Suppl Table S1) and the cell wall thickness measured by TEM (Fig. 6). For A cells, values were obtained from^22^.

Interestingly, the regions displaying the strongest BAM signals correspond to surfaces undergoing expansion. Prostrate filaments grow by elongation in the dome of the apical cell^33^, and E cells differentiate into R cells through radial swelling^35^. Cell expansion results from the combination of wall stress and of cell wall propensity to grow in response to this stress^36,37^. In order to discriminate between these two main factors – the wall stress on the one hand, and the cell wall plasticity on the other hand – with regard to the abundance of alginates, we calculated the wall stress along the filament and looked whether it is correlated to the BAM signals. Stress results from several biophysical components: while the wall stress increases with turgor, it decreases with the cell curvature and the cell wall thickness^36–38^. Therefore, we first measured the turgor in E and R cells using the technique of limit plasmolysis already used in *Ectocarpus*^22^ and found that it was similar in all both types (Suppl Table S1, t-test, P value = 0.407, see Methods for details). In a second step, we calculated the curvature (y-axis, Kappa s for the meridional curvature and Kappa theta for the circumferential curvature, in µm^−1^) for each cell type based on their average geometry (see Methods for details). We showed that the main changes in curvature were observed along the meridional axis, particularly along the A cell (Fig. 7). Using both the turgor and the curvature measurements, together with the thickness of the cell wall shown in Fig. 6, we calculated the wall stress as described in^39^ and^22^ (see Methods for details about the calculation). It showed that the highest wall stress (y-axis, 25.6 MPa) was at the extreme tip of the A cell, while it reached a basal level (3.3 MPa) in the shanks of A cells, similar to that calculated at the transverse junctions of all the other cell types ([3.1–3.8] MPa) (Fig. 7). Wall stress was slightly higher in the center of E and I cells ([4.7–6.1] MPa]) and culminated at 6.7 MPa in R cells.

This wall stress pattern reflects the pattern of alginate immunolocalisation: M-, MG- and G-rich blocks were present in the dome of A cells and in the shanks of R cells where the wall stress level is the highest, while shanks of A cells and E cells were almost never labelled. This suggests that alginates could be involved in the response to wall stresses above a given threshold, here estimated at ~5 MPa (Fig. 7).

### Alginates accumulate in response to hypotonic treatment

In order to test the hypothesis that alginates localise to areas subject to high wall stress, we cultivated *Ectocarpus* filaments in culture media with different osmolarities. *Ectocarpus* filaments were immersed in a hypotonic solution corresponding to half-strength sea water (550 mOsmol L^−1^) 24 h before immunolocalisation with the three BAM antibodies.

**Figure 8:**
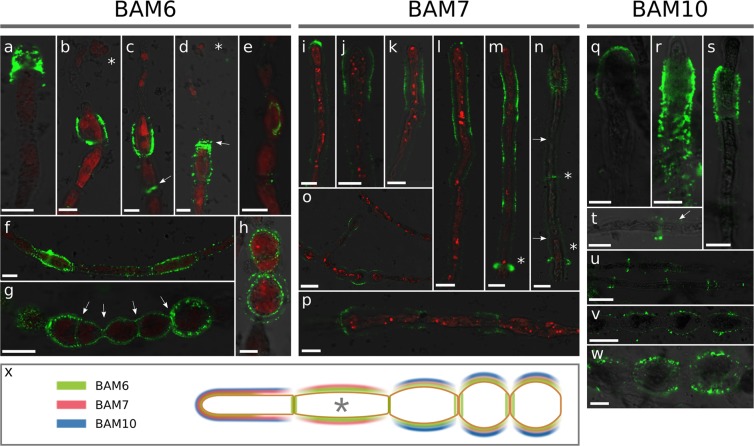
Alginate location in response to a hypotonic shock. Filaments were cultured in sea water (550 mOsmol.L^−1^), corresponding to half strength normal sea water, to increase cell turgor pressure. (**a**–**h**) BAM6 labelling: (**a**–**d**) Apex of filaments, showing either intact (**a**) or burst (**b**–**d**) A cells (asterisk: extruded chloroplast in burst A cells); (**e**) labelling of I cell side, also seen in E and R cells (not shown). (**f**) Portion of filament, showing that wider, I cells are more labelled than E cells. (**g**) R cells uniformly labelled, including transverse junctions (arrow). (**h**) Higher magnification of different labelled cell wall layers in R cells. (**i–p**) BAM7 labelling: (**i**–**k**) Apical cells; (**l**–**n**) Apical and sub-apical cells & discrete rings in E cells (asterisks); (**o**–**p**) Rare and weak labelling of R cells. (**q–w**): BAM10 labelling; (**q**–**s**) Apical cells; **(t**–**u**) E and I cells; (**v**–**w**) R cells. White arrows show transverse junction/wall. Micrographs are merged confocal stacks taken with three channels: green; FITC; red: chloroplast autofluorescence; grey: several lasers to simulate bright-field photos. Scale bar is 10 µm, except for o, u, v where it is 25 µm. (**x**) Summary of alginate mapping along the filament of *Ectocarpus* exposed to a hypotonic shock. Asterisk is for cases when the apical cell burst.

With BAM6 (M-rich), intact A cells displayed a more intense signal across the entire dome of A cells, in comparison to conditions corresponding to the natural sea water (NSW) (Fig. 8a). Interestingly, when A cells burst in response to the hypotonic shock, labelling was observed in the sub-apical cells (Fig. 8b–d), mainly at the external cell contour (Fig. 8b,c) but also at the junction between the A and the sub-apical cell (Fig. 8d, arrow) or even in more proximal junctions (Fig. 8c, arrow). The fact that these sub-apical cells have swollen in response to the hypotonic shock (note the round shape on Fig. 8b–d) strongly supports that they carry a higher load of wall stress, enhanced by the bursting of A cells. Therefore, MM alginates accumulate in the cells the most exposed to an increased pressure, which is either the A cell or the sub-apical E cell when the A cell has burst. Beside this main pattern, we also observed a peculiar labelling in the shanks of I cells, like an elongated crown spanning a single, lateral side (Fig. 8e). This might also display areas physically weakened because of the osmotic shock. Some significant signal was also observed in I and R cells (Fig. 8f,g) but not in E (non-sub-apical) cells (Fig. 8f). Interestingly, in contrast to normal sea water, hypotonic conditions caused labelling of transverse junctions (Fig. 8g) and of several cell wall layers in R cells (Fig. 2h), as observed with BAM7 in normal sea water.

In hypotonic conditions, the overall labelling of BAM7 was weaker than in normal sea water, and concentrated at the apex. In rare cases A cells showed a signal restricted to the dome (Fig. 8i), but the labelled area was usually extended to or exclusively in the shanks of these A cells (Fig. 8j,k), as well as along the sub-apical cells (Fig. 8l,m). In some cases, a fluorescent ring could be observed in more proximal positions, with no relation with the transverse cell junctions (Fig. 2m,n; asterisk). Signal was weak and rare in the other cell types (Fig. 8o,p).

The immunolocalisation pattern for BAM10 was most intense in the dome of A cells (Fig. 8q), extending down the shanks, making a very marked sleeve pattern in the sub-apical regions (Fig. 8r,s). Ring structures were observed in the central part of the filaments, either at transverse junctions (Fig. 8t) or independently of them (Fig. 8u). The shanks of some I and R cells were also labelled (Fig. 8v,w, respectively), in contrast to the NSW conditions. Moreover, no transverse boundaries between these cells were labelled, indicating a relocation of BAM10 from transverse boundaries to shanks in hypotonic conditions.

In summary, all antibodies displayed a new pattern corresponding to the shanks of A cells, or to sub-apical cells when they are directly exposed to the osmotic shock in the longitudinal axis (Fig. 8x). In addition, some “sleeve” patterns were newly observed, especially in cells the most exposed to a high tensile stress. This strongly suggests that alginates were deposited at these locations to reinforce the overstressed cell wall due to the hypotonic conditions.

**Figure 9:**
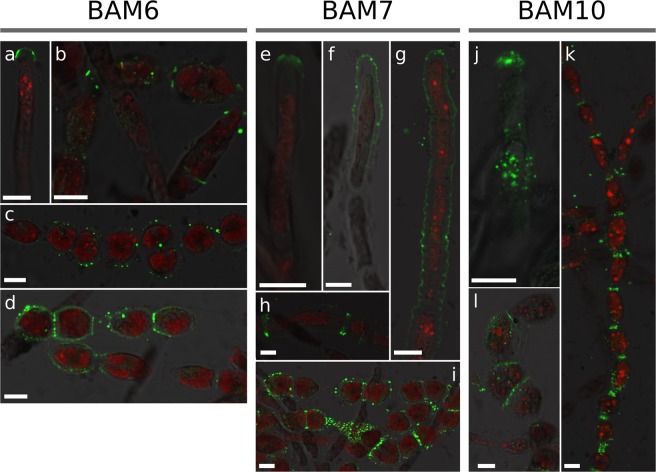
Alginate location in response to a hypertonic shock. Filaments were cultured in double strength sea water (2000 mOsmol L^−1^) to suppress the cell turgor pressure. (**a**–**d**) Immunolocalisation with BAM6: (**a**) Apical cell, (**b**) I cells, (**c**,**d**) R cells. (**e**–**i**) Immunolocalisation with BAM7: (**e**–**g**) Apical and sub-apical cells, (**h**) I cell bordered by transverse junctions with adjacent cells, (**i**) Group of R cells. (**j**–**l**) Immunolocalisation with BAM10: (**j**) Apical cell, (**k**) Portion of filament made of E and I cells, (**l**) R cells in the centre of a filament with a recently divided cell in the middle. Same confocal detection channels as in Fig. 8. Scale bar 10 µm.

In order to confirm this pattern, we performed the opposite experiment by culturing the filaments in a hypertonic solution (~2X natural sea water, i.e. 2000 mOsmol L^−1^) followed by immunolocalisation with the BAM antibodies after 24 h. In A cells, BAM6 labelling was rarely detected, and in positive cells the signal was restricted to the most distal regions (dome; Fig. 9a). In the other cell types, a weak signal was observed in transverse junctions between I and R cells (Fig. 9b), and a strong signal was seen uniformly distributed around R cells (Fig. 9c,d). BAM7 labelled very weakly the apical cells, either restricted to the tip (Fig. 9e), or over a larger region along the cell (Fig. 9f,g). Some transverse junctions were also labelled, with the double-ring characteristic of BAM7 observed in NSW (Fig. 9h). R cells were labelled mainly at these transverse junctions (Fig. 9i). The G-rich epitopes detected by BAM10 were displayed in a few A cells (Fig. 9j), and most signal was observed in the transverse junctions between E and I cells (Fig. 9k), and between R cells (Fig. 9l). Altogether, the three antibodies showed a heavily reduced signal in the apical cells, with the vast majority of signal being detected at the transverse junctions in hypertonic culture conditions.

In summary, these data show an overall trend: the more stressed the location along the filament, the strongest the signal of alginate immunostaining. This suggests that alginates could be more abundant in these areas, where they would participate in cell wall stiffening to resist the increase in wall stress. Therefore, they would control the intrinsic mechanical properties of the cell wall to cope with stress generated by cell shape or by rapid changes in osmolarities, and this functional relationship would be a priori independent from the growth process.

### Alginates co-localise with stiff areas

**Figure 10:**
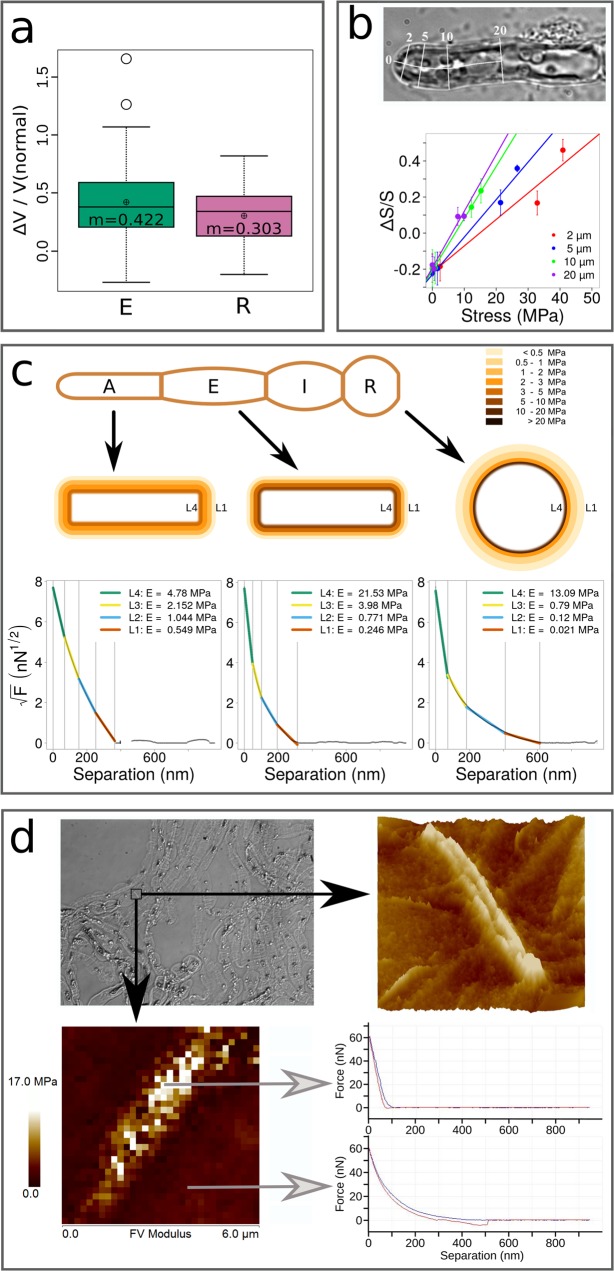
Stiffness along the filament. (**a**) Stiffness between E and R cells measured by dilatation/retraction experiments. Cell expansion was observed in response to immersion into fresh water. Plot represents the ratio of volume of E and R cells before and after immersion. Volumes were calculated from the cell dimensions, namely their length and width assuming that they are symmetrical. Measurements were carried out by ImageJ on bright field photos. n = 99 for E cells and n = 55 for R cells. T-test P value = 0.0106. (**b**) Stiffness in the dome measured by dilatation/retractation. The circumferential deformation of *Ectocarpus* apical cells was plotted as a function of the distance from the tip. Cells were subjected to inflation or retraction by transfer into hypo- or hypertonic sea waters respectively (see text for details). (Top) Relative circumferential deformation was measured at 2, 5, 10 and 20 µm from the tip. (Bottom) The deformation (calculated on a cell wall ring; ΔS/S) was plotted as a function of the local cell wall stress (σ_e_) calculated at each position after the deformation was stabilised. Normal condition (sea water ~1000 mOsmol L^−1^) is set to 0 (no deformation) for the four curves.  n = 63 cells for each curve. (**c**) Stiffness along the filament measured by nano-indentation using Atomic Force Microscopy. (Top) Scheme representing a filament stereotype indicating the position of the four cell types. (Middle) Schemes representing the section of A, E and R cell types with four virtual layers whose thicknesses were inferred from the slope of the force curve. Colour stands for the average Elastic Modulus calculated from the force curves (n = 6 for A cells, n = 7 for E cells, and n = 4 for R cells; Table 1), based on the Sneddon model (see Methods for details about calculation). (Bottom) Example of one force curve for each cell type. X-axis: distance of separation (nm); Y-axis: square root of force (nN^1/2^). (**d**) Stiffness at transverse junctions by AFM. Three junctions were imaged independently for three distinct filaments and gave similar results. Only one is shown here. (Top left) DIC image of extracted cell walls from filaments (see text for details). The transverse junction is framed. (Top right) Topography image of the transverse junction between two R cells, showing the relief of the central structure. (Bottom left) Corresponding elasticity map of 6 × 6 µm area (36 µm^2^) extracted from an array of 32 × 32 (1024) force curves. Force curves measured at the junction (top) and at the surrounding surface (bottom). Indentation: blue curve; retractation: red curve. Several acquisitions were carried out for each junction and gave similar data.

Stiffness of the cell wall was assessed along the filament using two complementary techniques. First, the propensity of in-plane (x/y) cell wall expansion in E and R cells was measured in living filaments. In order to avoid a limitation of cell expansion due to the adhesion of the filament to the substratum, we used free-floating filaments. The cell diameter in the centre of each cell (i.e. ~10–15 µm) was measured before (sea water = 1100 mOsm L^−1^) and 1 min after immersion into fresh water (0 mOsm L^−1^). Then, the expansion resulting from cell swelling was calculated as the percentage of the cell volume increase. Results showed that R cells increased their initial volume by 30% while E cells swelled by 42% (P value = 0.0106), meaning that in the in-plane axis, R cells are significantly stiffer than E cells (Fig. 10a).

The mechanical properties were similarly measured in the dome of A cells. However, in order to obtain more detailed data for this particular area, the relative radial deformation was calculated at 2, 5, 10 and 20 µm from the tip. In addition, two hypotonic (275 or 550 mOsm L^−1^) and two hypertonic (1900 or 2660 mOsm L^−1^) solutions were used. In parallel, the global in-plane wall stress (*σ*_*e*_) of the cell wall before and after deformation was calculated according to^39^. Strain curves plotted as a function of stress along the cell showed that the cell wall elasticity at 2 µm away from the tip was the lowest, while it increased gradually more distantly from the tip (10 and 20 µm positions; Fig. 10b). Therefore, these data display a negative gradient of circumferential deformability from the tip to the flanks of the A cell.

Interestingly, Terauchi *et al*.^22^ showed that the organisation of the cell wall in *Ectocarpus* up-right filaments is anisotropic in the z-direction, suggesting that the mechanical properties of the cell wall might be different in the different axes. Therefore, we used atomic force microscopy (AFM) on living filaments, to assess the stiffness of the cell wall in the z-axis, which is perpendicular to the in-plane axes considered above. Force curves obtained in the center of each cell type exhibited a non-linear behaviour, indicating that the Elastic Modulus was not constant through the cell wall width. For all curves, z values corresponding to the innermost part of the cell wall displayed a steeper slope, showing that the Elastic Modulus increased from the external to the internal part of the wall. To characterise these gradients in different cell types, we used a discretisation procedure, which consisted in optimally adjusting each profile to four linear segments (see Methods for details). We could thus assign limits to each of the corresponding cell wall parts, and compute a local Elastic Modulus value, using the Sneddon model. Results showed differences between A and R cells. In A cells, the outer layer (L1) was stiffer than in R cells, but the inner layer (L4) was softer (Fig. 10c). This makes the stiffness of cell wall in A cells more homogeneous, with an average Elastic Modulus ~3 MPa (Table 1). In contrast, R cells displayed a heterogeneous cell wall stiffness, with a gradient ranging from <1 MPa for the outer layer L1 to ~20 MPa for the inner L4 (Table 1). Measurements were performed in turgid cells, but as the turgor is similar in E cells and R cells (Suppl Table S1), it cannot account for the difference in Elastic Modulus observed between the cell types. Therefore, an increase in stiffness of the most recent, inner cell wall layer L4 is observed from the apex to more central positions in the filament. Altogether, the cell wall seems to stiffen both in the in-plane axis and in the z-axis as cells maturate from A to R cells.

**Table 1:** Elastic Modulus E of the four virtual cell wall layers L1–4 inferred from the force curves obtained by atomic force microscopy.

Cell type	A				E				I				R			
Sample size	n = 6 cells				n = 7 cells				n = 2 cells				n = 4 cells			
E (MPa)	L4	L3	L2	L1	L4	L3	L2	L1	L4	L3	L2	L1	L4	L3	L2	L1
**Mean**	3.84	2.48	1.43	0.46	7.72	4.23	1.99	0.24	18.91	7.29	3.11	0.54	16.88	2.77	0.60	0.21
**S.D**.	0.54	0.24	0.36	0.35	2.33	1.19	0.75	0.17	4.12	1.47	2.05	0.10	4.81	1.54	0.34	0.13
**Thickness (nm)**	0–83	83–176	176–264	264–345	0–69	69–147	147–217	217–286	0–41	41–92	92–155	155–219	0–55	55–129	129–261	261-404

For each cell, the force curve was optimally discretised into four linear parts. For each of the four cell wall layers, the mean (MPa) and SD are indicated, as well as the position (z-axis, or thickness) of the layer boundaries. L1 is the most external layer (i.e. the oldest), L4 the most internal one (i.e. the most recent).

AFM was also used to focus on the boundaries between adjacent cells. In some places, scanning and measurement of the mechanical properties showed a peculiar stiff structure, potentially doubled, protruding from the cell surface (Fig. 10d). This structure was reminiscent of the double-ring observed with BAM7 immunolocalisation (Fig. 3).

Altogether, both cell wall deformation and AFM showed that the cell regions immunolabelled by the BAM 6/7/10 antibodies are among the stiffest regions of the filament. These results support that alginates are necessary to maintain the cell wall stiff enough to resist to high wall stresses.

### Alginates contribute to cell wall resistance to stress

**Figure 11:**
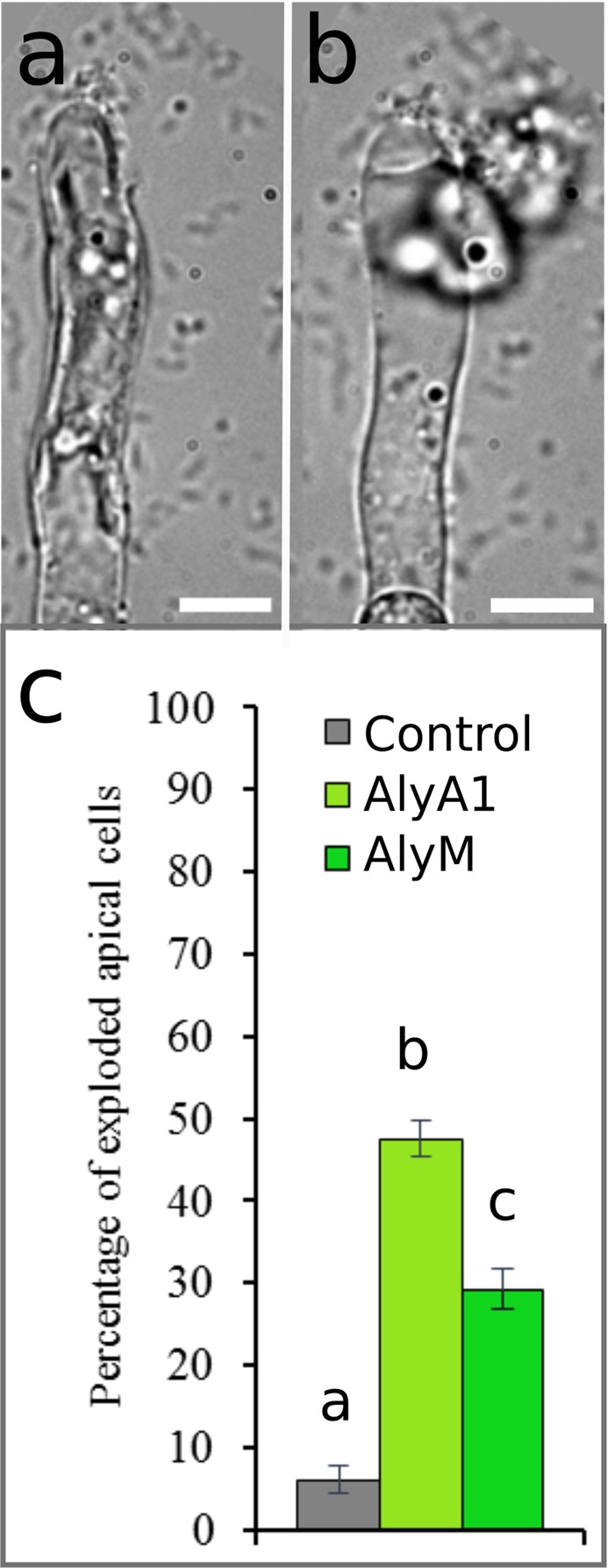
Alginates prevent tip bursting. (**a**) Apical cell before a hypotonic shock; (**b**) Apical cell after the hypotonic shock, showing where cell bursts. (**c**) Percentage of burst apical cells after 10 min of incubation with 3U mL^−1^ of AlyM (right column) or 3.2U mL^−1^ of AlyA1 (G-specific, middle column) in full-strength Artificial Sea Water (ASW, control: grey column). Error bar represents 95% confidence interval. n = 15 independent experiments representing more than 800 A cells. Bars with different letters are for significant differences. χ² test with p < 0.001. Scale bar 5 µm.

We tested the hypothesis that alginates contribute to the resistance of the cell wall to mechanical stress. The tip of apical cells are the primary location of cell bursting upon hypo-osmotic shock (Fig. 11a,b). We therefore added exogenous alginate lyases to our cultures and observed the effect of degrading alginates upon the capacity of the cell wall to resist elevated turgor pressure. Furthermore, in order to distinguish between the role of G-rich and M-rich alginates, we treated the filaments with two different enzymes, each specific for either GG blocks with the G-blocks-specific alginate lyase from *Zobellia galactanivorans* AlyA1, or  M-blocks with the alginate lyase AlyM (A1603, Sigma). Figure 11c shows that apical cells treated 10 min in AlyM and AlyA1 burst at a much higher frequency compared to control cells. The location of rupture is at the tip, as expected (Fig. 11b). The effect of AlyA1 was significantly higher than that of AlyM (*χ*^2^ test, p < 0.001). These results show that alginates ensure cell wall strength in the apical dome, and suggest that G-block alginates play a greater role in strengthening the cell wall than M-blocks.

## Discussion

### Role of alginates in managing the wall stress

In *Ectocarpus*, we have shown that alginates co-localise with the stiffest sites of cell walls along the developing prostate filament, which are also sites experiencing the highest wall stress. Furthermore, we provide evidence that alginates specifically contribute to the cell wall resistance to cell bursting. A role of alginates in brown algal tissue stiffness has long been suggested, based upon the differential composition of alginates extracted from tissues displaying different stiffness properties, notably the stipe compared to the blade, or wave-exposed blades compared to sheltered ones^40–44^. However, the analysis of whole tissues, which encompass hundreds of cells and specialised cell types, is hard to spatially dissect for specific structural roles. Here, we provide topological information at the cellular level within a whole organism with different cell types.

Within the developing prostrate filaments, R cells and the dome of A cells display the highest stiffness. While in mature filaments, R cells are at least 4 days old (~1 cell division every 12 hours, several E and I cells are present ahead of the group of R cells), A cells are continuously renewed via tip growth. Therefore, A cell stiffness cannot be accounted for by a process of cell wall maturation alone. *Ectocarpus* sporophytes develop on solid substrates (rocks or epiphytically on other algal thalli), similar to attaching disk or holdfast of Fucales and kelp (Order Laminariales) which grow inside rocky microstructures to get solid attachment^45,46^. A stiffer cell wall at the apex of tip-growing rhizoids might thus be an advantage to withstand strong mechanical stresses due to the compression and friction with such hard medium^47^. Furthermore, brown macroalgae are subject to high variations of salinity during tides, which impact the turgor pressure and extend the risk of tip bursting. Therefore, an increased stiffness of the cell wall in the apex would prevent too large deformation and further rupture. In contrast, a gradient of stiffness along the cell walls of the shanks would allow deformation, thereby maintaining the ability for the apical cell to balance turgor by volume change. Alteration of cell volume in response to turgor pressure has been observed in the cell wall of sieve elements in kelp, which can deflate in response to accidental drop on turgor^48^, reminiscent of radial mechanical properties of sieve elements in land plants^49^.

An interesting structural feature of *Ectocarpus* filament was revealed during this study. SEM displayed some protruding surface at the cell junctions. This structure was more frequently seen between adjacent R cells. G-rich alginates were more abundant at the same location, making their involvement in its formation very likely. Longitudinal TEM sections supported this hypothesis, as electron-dense material observed in osmium-treated *Ectocarpus* sections was shown to be alginate^21^. AFM showed that these structures displayed a high stiffness, suggesting that they could participate in the consolidation of the junctions, while the cells themselves would remain softer in comparison. However, the role of this peculiar structure fully remains to be elucidated.

Interestingly, the first alginate lyase of a brown algal origin was recently identified in the kelp *Saccharina japonica*^50^. This enzyme displays a higher activity for mannuronan substrates, and could thereby contribute to the re-organisation *in muro* of the cell wall intrinsic mechanical properties as cells experience variation in osmotic pressure or physical attacks by grazers and invasive intracellular pathogens and parasites.

### Do Mannuronan and Guluronan-rich alginates have different mechanical role *in muro*?

Alginates are an abundant class of components in the wall of brown algae^40,51^. They have been shown to reinforce the structural integrity of the wall in *Ectocarpus* sporophytic cells^21^. This effect is supposed to be mediated by the cross-linking of GG-blocks by calcium ions (Ca^2+^) forming the so-called “egg-boxes” junctions^16,52^. Indeed, it is a long-held opinion that GG-blocks of alginates provide most of the mechanical strength and rigidity of brown algal wall. At the thallus or organ level, some studies have observed a correlation between the apparent organ rigidity and the relative abundance of GG-blocks, generally quantified as the M/G ratio^41,42^. However, a lack of correlation between these two parameters was reported in other studies^28,43,53^. At the single cell level, a lower abundance of GG-alginate was detected in the terminal cells of *Adenocystis utricularis* (Ectocarpales), where most of growth occurs, and was supposed to give more “expandable” wall in these cells^54^. In this study, we showed that, while both alginate lyases caused a significant increase in tip bursting, digestion with an M-lyase caused a lower rate of apex bursting compared to G-lyase, suggesting that G-rich alginates are significantly present in the apical dome. Alginate polysaccharides are a mixture of the three types of blocks (MM, MG, GG), and thus are most likely present in all alginate polymers of the cell wall. Therefore, major distinct roles of M-rich or G-rich alginates are difficult to entirely resolve *in muro*.

Immunolocalisation revealed M-, MG- and G-enriched alginates in the apex of apical cells, suggesting that the growing wall is indeed not associated with higher M/G ratio. The work of Nagasato *et al*. showed that alginates were delivered to the plasma membrane by flat cisternae, which are vesicular bodies specific to brown algae^55^. Alginates are deposited in the cell wall first as pure MM-homopolymer^56,57^ and further converted to some extent into G unit *in muro* by irreversible epimerisation of M units on the C5 carbon^58^ by mannuronate-C5-epimerases (MC5E^57,59^). These enzymes are delivered to the cell wall through similar or parallel routes as alginates, but their presence in the brown algal cell wall has not been displayed yet. Although MC5Es were shown to be active in brown algal thalli^56,60^, it is not known in which cell compartments. However, protoplasts of the brown alga *Laminaria digitata* were shown to excrete MC5Es into the extracellular medium during the phase of wall-rebuilding, indicating that these enzymes probably act in the tissue apoplasts^57^. This hypothesis is supported by the recent genome-wide characterisation of the secretome of many brown algae, which identified MC5E as one of the major proteins secreted from *Ectocarpus* filaments, and even more significantly from *Saccharina* thalli^61^. Therefore, our results support that the formation of G units occurs simultaneously with the delivery of M units to the apex, most likely *in muro* during formation of the cell wall, and not later during cell wall maturation.

### A role of alginates in the control of growth?

Alginates were immunolocalised at positions where growth occurs, namely in the dome of the apical cells and in the shanks of I and R cells (rounding). Based on the knowledge in other organisms, a role of alginates in the softening of the cell wall prior to cell expansion during growth might be naturally called upon. In land plants^62–64^ and fungi^65^, the cell walls of tip-growing filamentous structures have been shown to be softer at the dome compared to the shanks, facilitating growth where the wall stress is lower. In the pollen tube, a stiffness gradient between the growing apical dome and the distal region of the tubular shanks has been displayed using nano-indentation^62,64,66^. It was shown to be primarily controlled by a gradient of de-methylesterification of homogalacturonan (HG) from the apical dome to the shanks^62,63^. This chemical gradient is itself controlled by pollen-specific pectin-methylesterases (PMEs), which activity is tightly regulated par PME inhibitors (e.g. PMEI) and other chemical factors^63,67,68^. Altogether, these factors maintain an extensible cell wall at the dome while rapidly strengthening the shanks to ensure transition to and maintenance of the tubular shape. In *V*a*ucheria terrestris*, an extensibility gradient was also found in the apical tip-growing part^69^.

Strikingly, in the present study we showed that the apical cell in *Ectocarpus* filaments displayed an inverted gradient of cell wall stiffness: the tip was stiffer than the shanks. This is an interesting example where the growth activity is dissociated from the intrinsic mechanical properties of the cell wall. Furthermore, we recently showed that the dome of *Ectocarpus* apical cell is subject to a high wall stress, due to an extremely thin cell wall at the tip (36 nm thick), which makes cell wall softening not required for growth^22^. However, this extreme cell wall thinness also makes the cell extremely prone to rupture. Consequently, mechanical reinforcement to prevent tip bursting and to ensure maintenance of cell integrity is required. Alginates would therefore ensure a high intrinsic mechanical resistance of the cell wall in this location. However, this role would be completely independent from the growth process, which relies only on the thinness of the cell wall^22^.

In conclusion, these results have important implications as the congruence between the chemical composition and the intrinsic mechanical properties are examined at the microscale level on an entire organism: the filamentous, uniseriate body of *Ectocarpus*. They also allowed to dissociate the intrinsic mechanical properties of the cell wall from the growth activity.

## Methods

### Ectocarpus culture

*Ectocarpus sp*. Ec32 (CCAP accession 1310/4; origin San Juan de Marcona, Peru) was cultured *in vitro* as described in^5^. In brief, immature haploid parthenosporophytes containing no up-right filaments, were grown in half-strength, Provasoli-enriched autoclaved natural sea water (NSWp, pH 8.7) in a controlled environment cabinet at 13 °C with a 14 h:10 h light-dark cycle (light intensity 29 μmol photon m^−2^ s^−1^). Culture medium was renewed every 10 days. For culture propagation, small tufts of filaments were transplanted and placed individually in new dishes. For immunolabelling experiments, deformability measurements and TEM observations, parthenosporophytes germinated on sterile glass coverslips or glass slides loaded in the bottom of Petri dishes. For the SEM experiments, sporophytes grew on a polycarbonate filtration membrane (nuclepore, diameter 13 mm, Cat N° 110406N, Whatman).

### Immunolocalization of alginates

Monoclonal antibodies (BAM6, BAM7 & BAM10) were raised and characterised as described in^29^. Immunolabelling procedures for *Ectocarpus* filaments are described in^70^, using anti-rat secondary conjugated to FITC. For each culture condition (Natural sea water, hypotonic and hypertonic media), experiments were performed on three cover slips covered with two-week old *Ectocarpus* prostrate filaments growing in separate Petri dishes. Observations of the FITC fluorescence were carried out using a TCS SP5 AOBS inverted confocal microscope (Leica).

### Scanning electronic microscopy (SEM)

SEM was performed as described in^23^. Briefly, *Ectocarpus* gametes were released and grew on a polycarbonate filtration membrane (Nuclepore, diameter 13 mm, Cat N° 110406 N, Whatman). After two weeks, parthenosporophytes were fixed in seawater with 3% paraformaldehyde for 1 h and then washed 10 min in a diluted Artificial Sea Water (ASW) ASW: H_2_O (3:2), ASW: H_2_O (2:3) and H_2_O, followed by successively dehydration steps in 30%, 50%, 70%, 90%, 2 times 95% and 3 times 100% EtOH. They were finally dried using a critical point dryer (Baltec CPD 030, Balzer), covered with a 25 nm thick gold layer and observed with a JEOL JSM 5200 scanning electron microscope.

### Transmission electronic microscopy (TEM)

Filaments were first fixed in 4% glutaraldehyde in 0.2 M cacodylate and 0.25 M sucrose, for 1 night at 4 °C. They were rinsed 10 min in 0.2 M cacodylate, 0.25 M sucrose and 0.225 M NaCl, 10 min in 0.2 M cacodylate, 0.15 M sucrose and 0.274 M NaCl, 10 min in 0.2 M cacodylate, 0.05 M sucrose and 0.325 M NaCl, and finally 10 min in 0.2 M cacodylate and 0.35 M NaCl, at RT. Filaments were then post-fixed in 1% OsO_4_ in 0.2 M cacodylate and 0.33 M NaCl, 1 h in dark. After rinsing 3 × 10 min in 0.2 M cacodylate in 0.35 M NaCl, filaments were dehydrated in an ethanol gradient: quickly in 30% EtOH, 50% 10 min, 70% 3 × 10 min, 90% 3 × 10 min and 100% 3 × 10 min. Filaments were embedded in Epon resin in BEEMS capsule, directly deposited upside-down upon the microscope slide. The resin was allowed to dry one night at 37 °C then one day at 60 °C. Following longitudinal sections were performed as described in^22^.

### Calculation of the wall stress profile in the four cell types

The A cell curvature was taken from^22^. E, I, and R cell were assumed to have truncated ellipsoid shapes, with extremal and central radius measured on micrographies. Meridional and circumferential stresses were respectively computed using the Hejnowicz formulae σ_s_ = T/(2 δ κ_θ_) and σ_θ_ = T/(2 δ κ_θ_) (2 − κ_s_/κ_θ_) where T stands for turgor, δ is the cell wall thickness and κ_θ_ and κ_s_ are respectively the circumferential and meridional curvatures^36^. The global stress was then computed as σ_e_ = [ν(σ_θ_ − σ_s_)² + (1 − ν)(σ_θ_² + σ_s_²)]^1/2^ where ν = (1 − K_s_/K_θ_)/2 is the flow coupling factor under the assumptions of the viscoplastic model^39^.

### Stress and surface deformation in A cells

Using ImageJ software, the longitudinal (axial) and circumferential deformations were measured as relative variations before and after filament immersion into hypotonic or hypertonic media (originally in articifial sea water at 1100 mOsm L^−1^), respectively in half-strength ASW diluted with deionized water (~550 mOsm L^−1^) or sea water saturated with sucrose (2660 mOsm L^−1^) . After 1 minute, the cell inflation/shrinking ceased, and was recorded on the same cells. Images were acquired with the Leica Application Suite software (LAS v2.2.1, Leica) and measurements were carried out using the ImageJ software at 2, 5, 10 and 20 µm away from the tip of A cells. The cell axial variation appeared to be negligible (~1 µm out of 25 µm total length). Consequently, the four positions along the apical cells (x) were considered to be the same before and after the cell inflation/shrinking. For each of these four positions, the global stress (σ_e_) was computed as indicated above assuming the cell wall thickness gradient modelled by^22^. To calculate the surface deformation at each of these positions, we approximated the dome geometry by a half ellipsoid with a long radius (along the main axis of the cell) of 8 µm. For axial abscissae (origin at the tip of the dome) *x* of 2 and 5 µm, we computed the surface S, as the area of a strip of cell wall surrounding the cell at *x* ± 10 nm, by summing 100 small surfaces of truncated cones with δ*x* = 0.2 nm. The surfaces around *x* = 10 and 20 µm were computed as the surface of a cylinder, as they do not belong to the dome. Surfaces in normal (S_0_) and in test condition (S_1_) were computed the same way, and the deformation factors S_1_/S_0_ obtained for cells in similar conditions were averaged by computing their geometric mean. For the graphic representation we plotted the relative surface deformation ΔS/S_0_ = (S_1_/S_0_)^−1^. For each condition, a batch of 3 to 32 apical cells per level of osmotic stress were recorded and measured. The means ± S.D. were calculated and tested by Welch-corrected t-Student tests.

### Atomic force microscopy

Acquisition of force curves were carried out on *Ectocarpus* filaments growing on glass slides, immersed in a Petri dish filled with sea water. Force indentation curves were acquired using ScanAsyst fluid cantilevers (Bruker) with a spring constant of approximately 1.5 N/m, and an indenter with a triangular shape (Scanasyst). Cantilever was calibrated by measuring deflection sensitivity and spring constant. The deflection sensitivity was determined by recording a force curve on a hard surface (glass slide) in seawater. The spring constant was then measured using the thermal tune method in air, which consists in the determination of the resonance frequency of the cantilever. A maximum load of 60 nN was used. Cartographies of elasticity were obtained by fitting the curves with the Sneddon model. Because AFM is a surface technique, obtaining information about the thickness of the cell wall requires complex calculation. Cross-sections of the cell wall would provide mechanical data of the different layers, but only in an axis parallel to the surface of the cell and not in its depth. Instead, data processing allowed the discretisation of the cell wall into slices of homogeneous stiffness in order to produce a simplified view allowing comparison between the different cell types. Technically, each curve √F = f(∆z) was optimally adjusted using R^71^ to four straight lines in two steps: (1) the central breakpoint was chosen among the experimental points, as the point for which the sum-of-square distances between the observations and the two-segments linear regression model was minimal. (2) the same procedure was applied for each of the two resulting sub-plots, resulting in four optimally adjusted linear segments. The inferred z values were considered as region boundaries. Local average stiffness (in MPa) for each region was estimated as the square of the slope multiplied by the constant factor K = π(1 − ν²)/(2 tan(α)) where ν = 0.5 is the Poisson ratio and α = 18° is the half cone angle. Results of calculation are shown in Table 1. Topography images (transverse junctions) were acquired *in vivo* in seawater with a SNL10, 0.32, radius = 2 nm.

### Turgor assessments by limit plasmolysis

Technique used was previously described in details in^22^. Briefly, it consists in immersing filaments in a series of media with different osmotic potentials, prepared from artifical seawater in increasing concentration of sucrose. Solution in which cells stop shrinking is considered the equilibrium between the external and internal concentrations. Final internal concentration was calculated by taking into account the variation in cell volume due to the shrinking.  n= 9 independent experiments, with n > 15 cells for each tested solution. P value = 0.407.

### Measuring bursting rates of apical cells in response to enzymatic treatments

The M-specific alginate lyase (AlyM) was purchased from Sigma (A1603). The stock solution was prepared at 75.08 mg mL^−1^ (≥750.8 U mL^−1^) in Phosphate Buffered Saline (PBS). The G-specific alginate-lyase corresponds to AlyA1 from the marine bacteria Z*obellia galactanivorans*^72^ and was produced in the lab. The stock solution of AlyA1 was prepared at 15 mg mL^−1^ (3225 U mL^−1^) in 100 mM Tris, 200 mM NaCl, pH 7.5 and was filtrated through a 0.20 µm membrane filter. To treat living filaments, enzymes were directly diluted into the culture medium at the final concentration of 0.3 mg mL^−1^ (≥3U mL^−1^) for AlyM, and 0.015 mg mL^−1^ (3.2 U mL^−1^) for AlyA1. The effect on tip bursting was observed in bright field microscopy 10 min after enzyme addition. Percentage of burst apex was calculated as (number of burst apical cells)/(total number of apical cells). Results from at least four independent experiments were gathered for each condition. Difference between conditions was tested by applying *χ*^2^ test.

## Supplementary information

Supplementary Table S1

## Data Availability

All data are available on request.
