# Supplementary material for: Alginates along the filament of the brown alga Ectocarpus help cells cope with stress
Source: Sci Rep. 2019 Sep 10;9:12956. doi: 10.1038/s41598-019-49427-z (PMC6736953; doi:10.1038/s41598-019-49427-z)
Supplement: Supplementary file 1 — Supplementary Table S1 [file 41598_2019_49427_MOESM1_ESM.pdf]

Title: Alginates along the filament of the brown alga *Ectocarpus* help cells cope with stress.

Authors: Hervé Rabillé[1], Thomas A Torode[2], Benoit Tesson[3], Aude Le Bail[1,*], Bernard Billoud[1], Elodie Rolland[1], Sophie Le Panse[4], Murielle Jam[5], Bénédicte Charrier[1*]

Supplementary information

**Cell type**	**Correction factor**	**Corrected turgor (mosmoles)**										
		**Replicate**								**Mean**	**S.D.**	**t-test p-value**
		1	2	3	4	5	6	7	8			
E	0.7	1465.6	1160.8	953.6	1177.6	1177.6	1105.5	1121.2	1122.9	1160.6	142.7	0.4
R	0.6	1622.7	1220.7	1002.8	1238.4	1238.4	1162.5	1166.8	1180.9	1229.2	176.1	

Supplementary Table S1 : Turgor measured in E and R cell types, using the « limit plasmolysis » technique.
